# DEBIO 1143, an IAP inhibitor, reverses carboplatin resistance in ovarian cancer cells and triggers apoptotic or necroptotic cell death

**DOI:** 10.1038/s41598-018-35860-z

**Published:** 2018-12-14

**Authors:** Benoît Thibault, Ludivine Genre, Augustin Le Naour, Clothilde Broca, Eliane Mery, Grégoire Vuagniaux, Jean Pierre Delord, Norbert Wiedemann, Bettina Couderc

**Affiliations:** 1 Institut Claudius Regaud – IUCT Oncopole, University Toulouse III, Toulouse, France; 2 Debiopharm International SA, CH-1002 Lausanne, Switzerland

## Abstract

The poor prognosis of ovarian cancer (it is the leading cause of death from gynecological cancers) is mainly due to the acquisition of resistance to carboplatin. Among the possible resistance pathways, resistance to apoptosis and especially the overexpression of inhibitor of apoptosis proteins (IAP) cIAP1 and X-linked IAP (XIAP), have been implicated. DEBIO 1143, a SMAC (second mitochondria-derived activator of caspase) mimetic, belongs to a new class of targeted agents currently being evaluated in clinical trials, which activate apoptotic cell death and block pro-survival signaling in cancer cells. Here, we demonstrate that DEBIO 1143 *in vitro* inhibits the cell viability of two carboplatin-sensitive cell lines (IGROV-1 and A2780S) as well as three carboplatin-resistant cell lines (A2780R, SKOV-3 and EFO-21). Of note, DEBIO 1143 is able to reverse resistance to carboplatin by inducing cell death either by apoptosis or necroptosis depending on the cell lines. To identify a biomarker able to predict the sensitivity of the cell lines to DEBIO 1143 treatment we analyzed the expression of the DEBIO 1143 targets cIAP1 and XIAP, and one of their downstream targets, caspase 9. These proteins did not constitute a marker of DEBIO 1143 sensitivity/resistance. Importantly, we confirmed these findings *in vivo* in SKOV-3 xenograft models where DEBIO 1143 highly potentiated carboplatin treatment.

## Introduction

Ovarian cancer management remains a great challenge. This cancer is the leading cause of gynecological cancer death and the fourth-leading cause of cancer death in women^1^. 70% of patients are diagnosed at advanced stages (III and IV); the 5-year survival rate for these patients is only 30%^2^. The reference treatment is debulking surgery followed by chemotherapy combining carboplatin and paclitaxel. Despite an initial clinical response in most patients (70 to 80%), recurrence frequently occurs, with acquired resistance to carboplatin^2,3^. There have been few improvements in the management of ovarian cancer for 20 years. The addition of an antiangiogenic treatment (bevacizumab) to the chemotherapy backbone therapy at the first recurrence^4^ and more recently the addition of an anti-PARP (Poly (ADP-Ribose) Polymerase) maintenance treatment (Olaparib) for platinum-sensitive relapsed patients^5^, have achieved clinical improvements. However, for most patients with ovarian cancer there is still a crucial need to develop new therapies that can the carboplatin resistance that ultimately occurs.

Carboplatin treatment of cancer cells induces apoptosis, a highly regulated cell death program. The balance between activators and inhibitors of this pathway may contribute to both innate and acquired chemoresistance, especially in ovarian cancer^6,7^. Tumor cells can resist apoptosis by, among other processes, increasing the expression of proteins blocking pro-apoptotic pathways. Overcoming the fundamental mechanisms of cancer resistance and survival, and activating cancer cell death through apoptosis, is a focus of current trends in cancer research and drug development. One novel therapeutic approach is the development of small molecule drugs that mimic SMAC (second mitochondria-derived activator of caspase), a pro-apoptotic mitochondrial protein that is an endogenous inhibitor of a family of cellular proteins called the inhibitor of apoptosis proteins (IAPs). IAPs regulate apoptosis and cancer cell survival and are considered to be part of the last line of defense for cancer cells against cell death by apoptosis. Among the eight IAP members that have been identified in mammalian cells, cIAP1 and cIAP2 interact with tumor necrosis factor receptor-associated factor 2 (TRAF2), blocking the formation of the caspase 8 activation complex and thereby inhibiting TNF receptor-mediated apoptosis^8–10^. X-linked IAP (XIAP), on the other hand, binds to and antagonizes three caspases, including two effectors, caspase 3 and 7, and an initiator, caspase 9, thus blocking both intrinsic and extrinsic apoptosis (mitochondria-mediated and death receptor-mediated apoptosis)^8,11^. Sui *et al*. state that IAPs are frequently deregulated in ovarian cancer^12^. Several publications have highlighted the involvement of XIAP in chemoresistance acquisition in various cancers, including ovarian cancer^13–15^. We have previously shown that ovarian cancer cells co-cultured with cells from their microenvironment, such as mesenchymal stromal cells acquired resistance to apoptosis due to activation of the Akt pathway and stabilization of XIAP^16^. Thus, exploiting IAP-caspase interaction may be a logical strategy for anti-cancer drug development and sensitization to carboplatin.

Several studies have reported that targeting XIAP, and more generally all IAP proteins, enhanced cell death *in vitro* and sensitized platinum-resistant ovarian tumor cells^14,15,17^. *In vivo*, XIAP inhibition in ovarian tumor models inhibits tumor growth and increases the survival rate in mice^18,19^. Together, these data suggest that targeting IAPs may be of therapeutic interest by sensitizing ovarian tumors to carboplatin.

Several “SMAC mimetic” molecules are being developed as cancer therapies aiming to sensitize tumor cells to apoptosis by antagonizing cIAP1/2 and XIAP activity. These SMAC mimetics can induce rapid degradation of cIAP1 and cIAP2 in cells; antagonize the functions of XIAP in functional assays and display anti-tumor effects in ovarian cancer and other *in vivo* mouse models when administrated alone or in combination with TNF-α, TRAIL (TNF-related apoptosis-inducing ligand), radiotherapy, or chemotherapies such as cisplatin or paclitaxel^20–23^, and more recently immunotherapies. The antitumor effect of SMAC mimetics when combined with immunotherapies is due to IAP-dependent regulation of NF-κB signaling pathways having a major impact on the function of the immune system, affecting both innate and adaptive immunity^24,25^. Thus, IAPs regulate the function of several immune cell types relevant for antitumor immune responses including antigen-presenting cells, lymphocytes, and natural killer cells, and IAP inhibition translate into marked enhancement of the efficacy of immune checkpoint inhibitors^26,27^.

DEBIO 1143 (AT-406, SM-406) is an orally active SMAC mimetic targeting cIAP1, cIAP2 and XIAP^28^. This SMAC mimetic showed a potent anti-tumor efficacy, alone or in combination with chemotherapies, in breast and ovarian xenograft mouse models and is highly effective in inducing apoptosis in those tumors^29,30^. DEBIO 1143 is currently in phase II clinical trials for the treatment of solid human tumors^31^, with three ongoing phase II trials. Notably, in a phase I study, signs of activity were observed in patients with ovarian cancer in combination with carbotaxol. This combination is currently being further studied in patients with newly diagnosed epithelial ovarian cancer (EOC) in the neoadjuvant setting (prior to interval debulking surgery) in a multicenter, double-blind, randomized, placebo-controlled phase II study (EudraCT Identifier 2015-005137-42)^32^. However, little is known about the mechanism underlying the anti-tumor efficacy of DEBIO 1143 in ovarian tumor cells and the cell death pathways involved still require deciphering.

In this study, we tested the *in vitro* cytotoxicity of DEBIO 1143 in two carboplatin-sensitive cell lines (IGROV-1 and A2780S) and three carboplatin-resistant cell lines (A2780R, SKOV-3 and EFO-21). While *in vitro* DEBIO 1143 had no cytotoxic effect as monotherapy, it was able to reverse carboplatin resistance. The mechanism involved cell death by either apoptosis or necroptosis, depending on the cell line. We show that DEBIO 1143 is able to potentiate carboplatin treatment in human ovarian cancer cells that are resistant to either apoptosis and/or necroptosis. To identify a biomarker able to predict the sensitivity of cell lines to DEBIO 1143 treatment, we analyzed the expression of the DEBIO 1143 targets cIAP1 and XIAP, and one of their downstream targets, caspase 9. These proteins did not constitute markers of DEBIO 1143 sensitivity/resistance. *In vivo* results demonstrate that DEBIO 1143 constitutes a potent anti-tumor agent as monotherapy and is able to overcome resistance to carboplatin.

## Results

### DEBIO 1143 reverses carboplatin resistance *in vitro*

**Figure 1:**
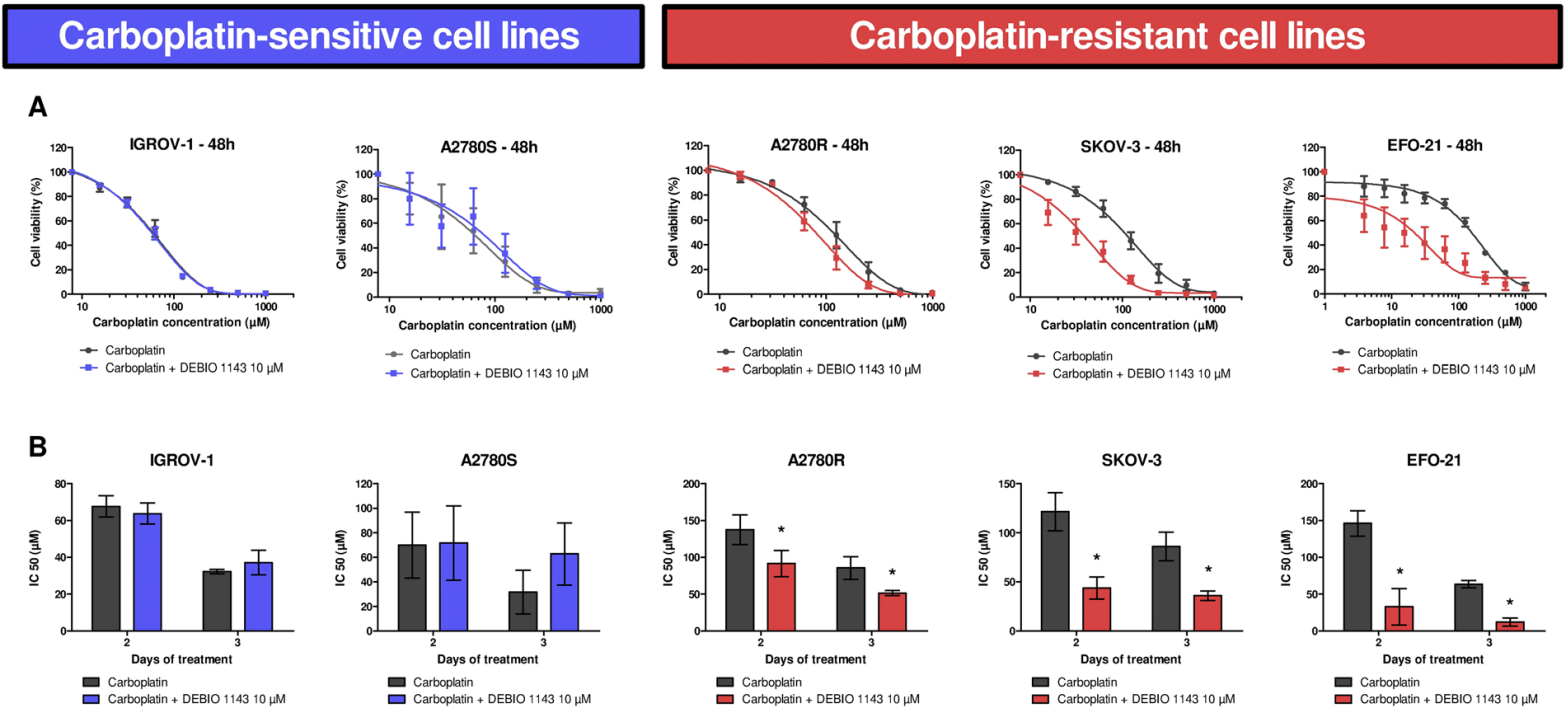
*In vitro* inhibition of HOAC viability by carboplatin alone, or in combination with DEBIO 1143. HOAC (OVCAR-3, IGROV-1, A2780S, A2780R, SKOV-3, and EFO-21) were treated with increasing concentrations of carboplatin alone (from 1000 µM to 15.625 µM by 2-fold dilution steps) or in combination with DEBIO 1143 (10 µM) 24 h after seeding. (**A**) 48 h after treatment, cell viability was determined by a colorimetric assay using WST-1. The negative control (non-treated) of each condition corresponds to the 100% cell viability. (**B**) The IC50 of carboplatin after 48 h or 72 h of treatment was determined for each cell line (Mean +/− SEM, *p < 0.05, n = 3).

We first tested the cytotoxic effects of carboplatin on five HOAC (Human ovarian adenocarcinoma cell): IGROV-1, A2780S (wild type), A2780R (A2780S after long-term carboplatin treatment), SKOV-3, and EFO-21. We treated these cells with increasing concentrations of carboplatin and evaluated cell numbers after 48 h and 72 h using a colorimetric assay. The carboplatin induced a concentration-dependent decrease in viable cells (Fig. 1). We determined the relative IC50 of carboplatin after 48 h and 72 h and concluded that IGROV-1 and A2780S cells are carboplatin-sensitive (IC50 values around 70 µM) and A2780R, SKOV-3, and EFO-21 cells are carboplatin-resistant (IC50 values > 100 µM) (Fig. 1).

To evaluate the capacity of DEBIO 1143 to sensitize cells to carboplatin, we treated our HOAC with increasing concentrations of carboplatin in combination with 10 µM of DEBIO 1143. DEBIO 1143 was not able to enhance the sensitivity of carboplatin-sensitive cells to carboplatin after 48 h or 72 h of treatment (Fig. 1). However, we observed that DEBIO 1143 significantly sensitized carboplatin-resistant cells to carboplatin with a 1.5 (A2780R) to 5.2-fold (EFO-21) reduction of the IC50, depending on the cells. Moreover, after 72 h of treatment, DEBIO 1143 totally restored the carboplatin sensitivity of A2780R to levels comparable with its parental cell line A2780S (Fig. 1B). DEBIO 1143 alone induced a very small decrease in viable cells starting at high concentrations, with the exception of A2780S and EFO-21 cells, which showed a higher sensitivity to the compound (Supplementary Fig. 1). Thus, DEBIO 1143 potently reversed the chemoresistance of carboplatin-resistant cell lines.

### DEBIO 1143 increases carboplatin-induced apoptosis in the carboplatin-resistant cell lines A2780R and SKOV-3, but necroptosis in EFO-21

Apoptosis is the most common cell death program induced by carboplatin. Moreover, DEBIO 1143 targets IAPs, which constitute key inhibitors of this cellular pathway. We therefore studied the capacity of DEBIO 1143 alone, or in combination with carboplatin, to induce apoptosis.

We treated our HOAC with carboplatin (IC50 of each cell line after 48 h of treatment) or DEBIO 1143 (10 µM), or with a combination of both treatments. After 48 h of treatment, we quantified early apoptotic cells according to an Annexin-V/PI assay.

**Figure 2:**
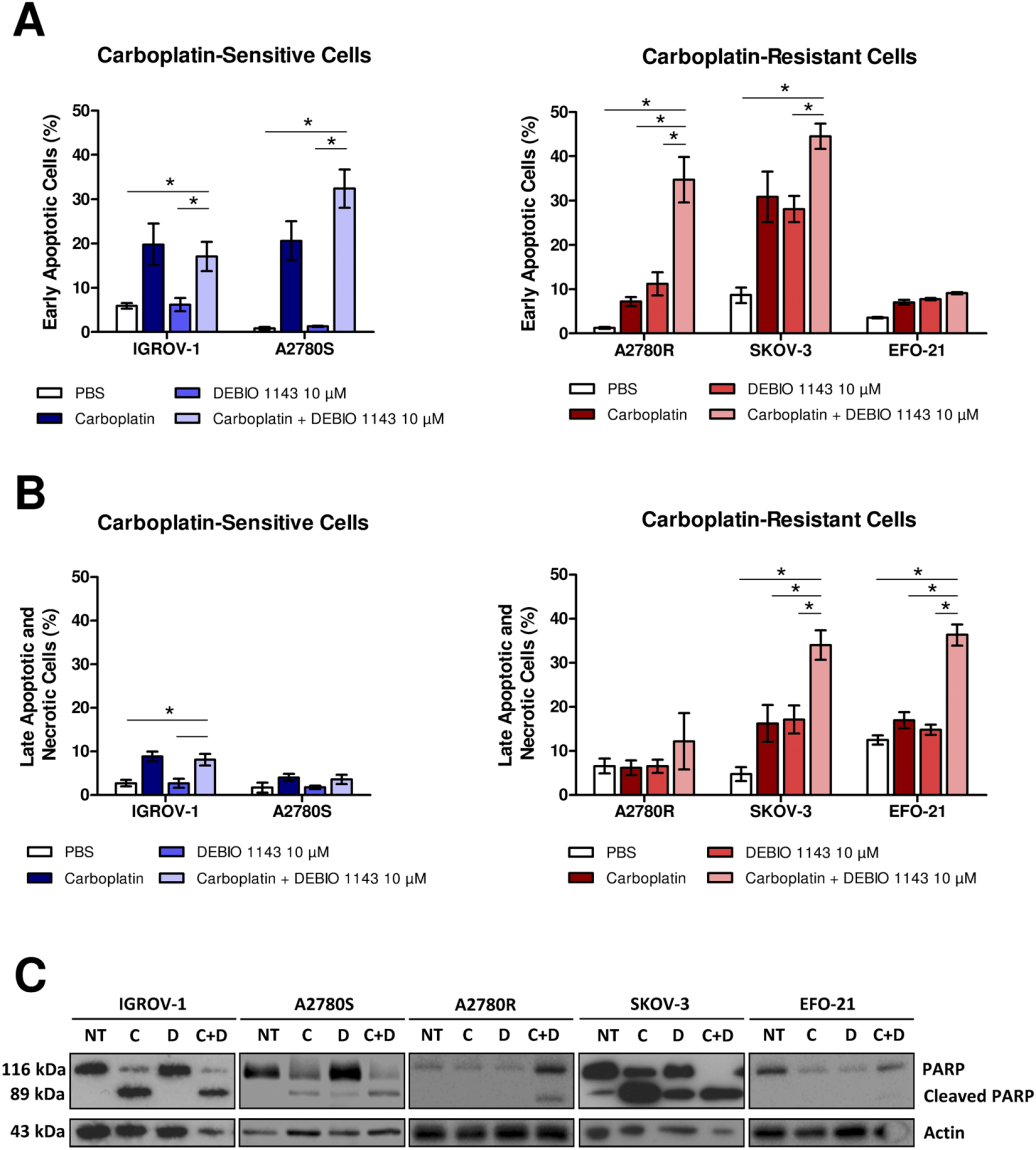
*In vitro* induction of apoptosis in HOAC by DEBIO 1143 alone or in combination with carboplatin. HOAC (OVCAR-3, SKOV-3, A2780S, and A2780R) were treated with carboplatin (IC50 after 48 h of treatment for each cell line), DEBIO 1143 (10 µM) or a combination of both treatments. The negative control corresponds to non-treated cells. (**A**,**B**) 48 h after treatment, cells were stained with a FITC-Annexin V/PI apoptosis detection kit. FITC-Annexin staining and PI incorporation were measured in cells with a FACS Calibur flow cytometer and analyzed with Cell Quest software. (**A**) Early apoptotic cells correspond to the Annexin V positive and PI negative population. (Mean +/− SEM, *p < 0.05, n = 3). (**B**) Late apoptotic and necrosis cells correspond to the Annexin V positive and PI positive population. (Mean +/− SEM, *p < 0.05, n = 3). (**C**) Western blots were used to analyze proteins extracts for the expression of PARP and cleaved PARP.

Carboplatin greatly enhanced the percentage of early apoptotic cells (Annexin-V^+^/PI^−^) in carboplatin-sensitive cells (IGROV-1 and A2780S) and in one carboplatin-resistant cell line, SKOV-3 (Fig. 2A). DEBIO 1143 alone did not induce apoptosis in our cell lines, except in SKOV-3 cells, which showed a 30% increase in of early apoptotic cells. The combination of DEBIO 1143 and carboplatin had no significant effect on carboplatin-sensitive cells compared to carboplatin treatment alone, but greatly increased the percentage of early apoptotic cells in carboplatin-resistant A2780R and SKOV-3 cells compared to both treatments alone. Surprisingly, no treatment induced apoptosis in EFO-21 cells, whereas the carboplatin/DEBIO 1143 combination was highly cytotoxic in these cells (Fig. 2A, Supplementary Fig. 2A). However, this combination induced a significant increase in Annexin-V^+^/PI^+^ EFO-21 cells, indicating that DEBIO 1143 in association with carboplatin could trigger necrosis or necroptotic cell death. SKOV-3 cells presented the same pattern but to a lesser extent.

We studied PARP cleavage, which is associated with early apoptotic cells (Fig. 2C), and found that the increased cleavage induced by the carboplatin/DEBIO 1143 combination occured mostly in carboplatin-resistant A2780R and SKOV-3 cells. In EFO-21 cells, no treatment induced any PARP cleavage confirming that the DEBIO 1143 could be responsible for an apoptosis-independent cell death in this cell line. In addition to apoptosis, cIAP1 and cIAP2 have important roles in the necroptotic death pathway^33^, a caspase-independent cell death pathway mainly regulated by receptor-interacting protein kinase 1 and 3 (RIPK-1 and RIPK-3) that shares common properties with apoptosis and necrosis. McCabe *et al*. found that the induction of necroptosis was common in ovarian cancer^34^.

**Figure 3:**
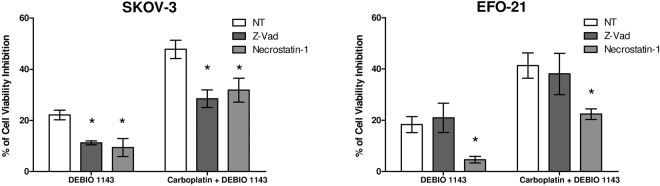
*In vitro* blockade of apoptosis or necroptosis in HOAC treated with DEBIO 1143, alone or in combination with carboplatin. HOAC (SKOV-3 and EFO-21) were pre-treated for 2 h or not with Z-Vad 20 µM or necrostatin-1 50 µM, then treated with DEBIO 1143 (10 µM) or a combination of carboplatin (IC50 of each cell line after 48 h of treatment) and DEBIO 1143 (10 µM). 24 h after treatment, cell viability was determined by a colorimetric assay using WST-1. Cell viability inhibition percentage was determined compared to the negative control (no drug, NT) condition. (Mean +/− SEM, *p < 0.05, n = 3).

We pre-treated EFO-21 and SKOV-3 cells with Z-Vad (pan-caspase inhibitor) or Necrostatin-1 (RIPK-1 inhibitor) and treated HOAC with DEBIO 1143 alone or in combination with carboplatin for 24 h (Fig. 3). In SKOV-3 cells, both Z-Vad and Necrostatin-1 inhibited nearly half of the cell viability inhibition induced by DEBIO 1143 alone or in combination with carboplatin, indicating activation of apoptosis but also of necroptosis in these cells. In EFO-21 cells, Z-Vad had no effect, confirming an apoptosis-independent pathway. However, Necrostatin-1 strongly inhibited cell viability inhibition induced by DEBIO 1143 alone and partially inhibited this parameter when DEBIO 1143 was combined with carboplatin.

In summary, these results indicate that DEBIO 1143, as a single agent or in combination with carboplatin, can induce cell death in carboplatin-resistant cells lines *via* two distinct mechanisms, by either sensitizing cells to carboplatin-induced apoptosis, or by inducing necroptotic cell death.

### DEBIO 1143 induces degradation of cIAP1 but not XIAP in both compound-sensitive and resistant human ovarian adenocarcinoma cell lines

**Figure 4:**
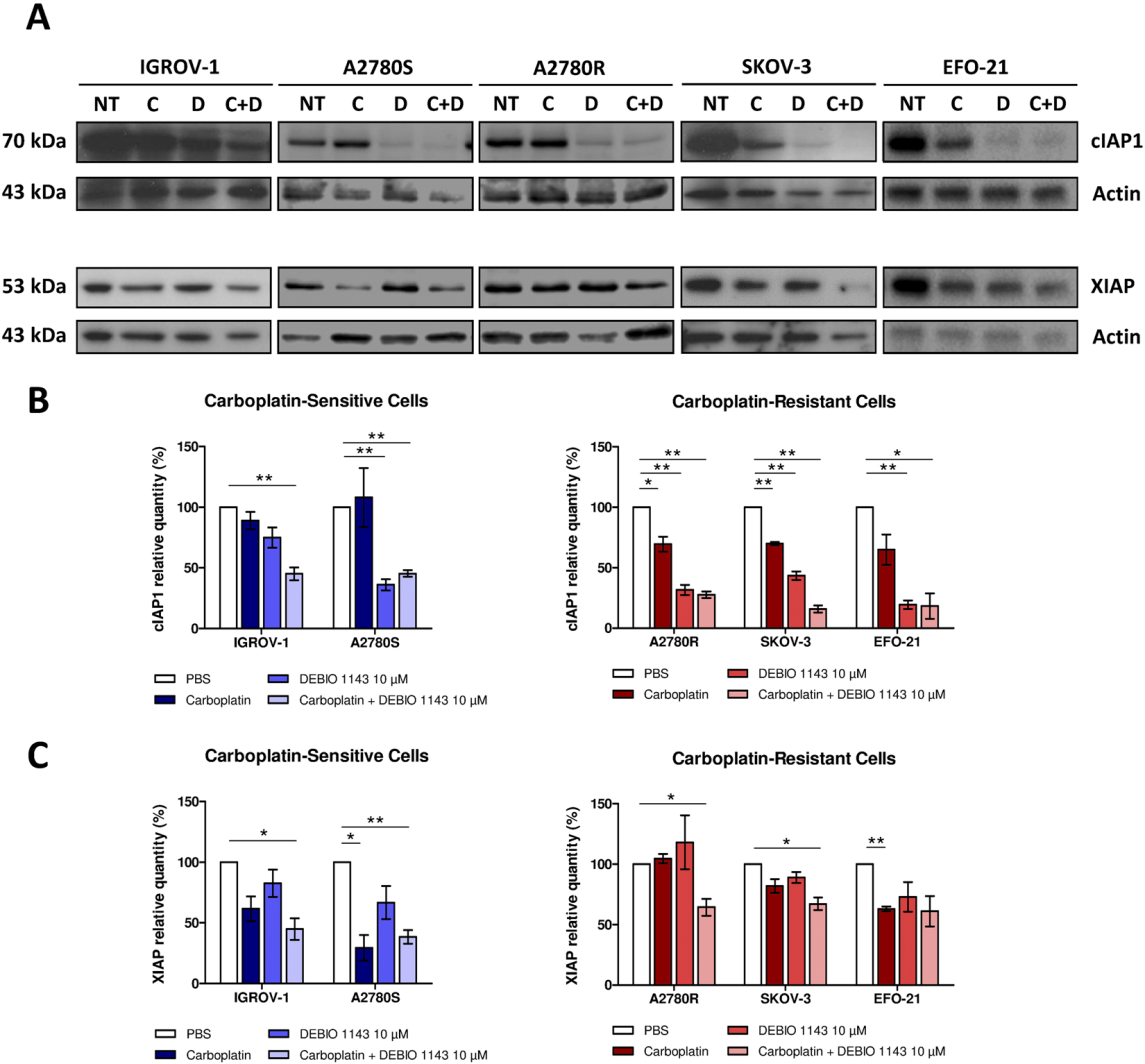
Effects of DEBIO 1143, alone or in combination with carboplatin, on the expression of targets. (**A**) HOAC (IGROV-1, SKOV-3, A2780S, A2780R, and EFO-21) were treated with carboplatin (IC 50 after 48 h of treatment for each cell line) (**C**), DEBIO 1143 (10 µM) (**D**) or a combination of both treatments (C+D). The negative control corresponds to non-treated cells (NT). Western blots were used to analyze proteins extracts for the expression of cIAP1 and XIAP. (**B**) cIAP1 and (**C**) XIAP proteins were quantified in 3 independent experiments and represented as relative quantities compared to the negative control. (Mean +/− SEM, *p < 0.05, **p < 0.01, n = 3).

DEBIO 1143 was designed to target and trigger the proteasomal degradation of IAPs. Thus, we decided to study cIAP1 and XIAP levels using Western blots in HOAC treated with carboplatin, DEBIO 1143, or the combination of both drugs, to find a potential marker of DEBIO 1143 sensitivity/resistance (Fig. 4).

DEBIO 1143 alone, or in combination with carboplatin, triggered a strong reduction of cIAP1 level in the five cell lines, regardless of their sensitivity/resistance to the compound. However, DEBIO 1143 alone had no effect on XIAP level in most cell lines, with the exception of EFO-21 cells, where the compound induced detectable reductions in XIAP levels. In addition, carboplatin reduced XIAP protein expression in IGROV-1, A2780S, SKOV-3, and EFO-21 cells with variable efficiency.

Thus, cIAP1 and XIAP are not reliable markers of DEBIO 1143 sensitivity/resistance.

### DEBIO 1143 efficacy in HOAC does not depend on caspase 9

**Figure 5:**
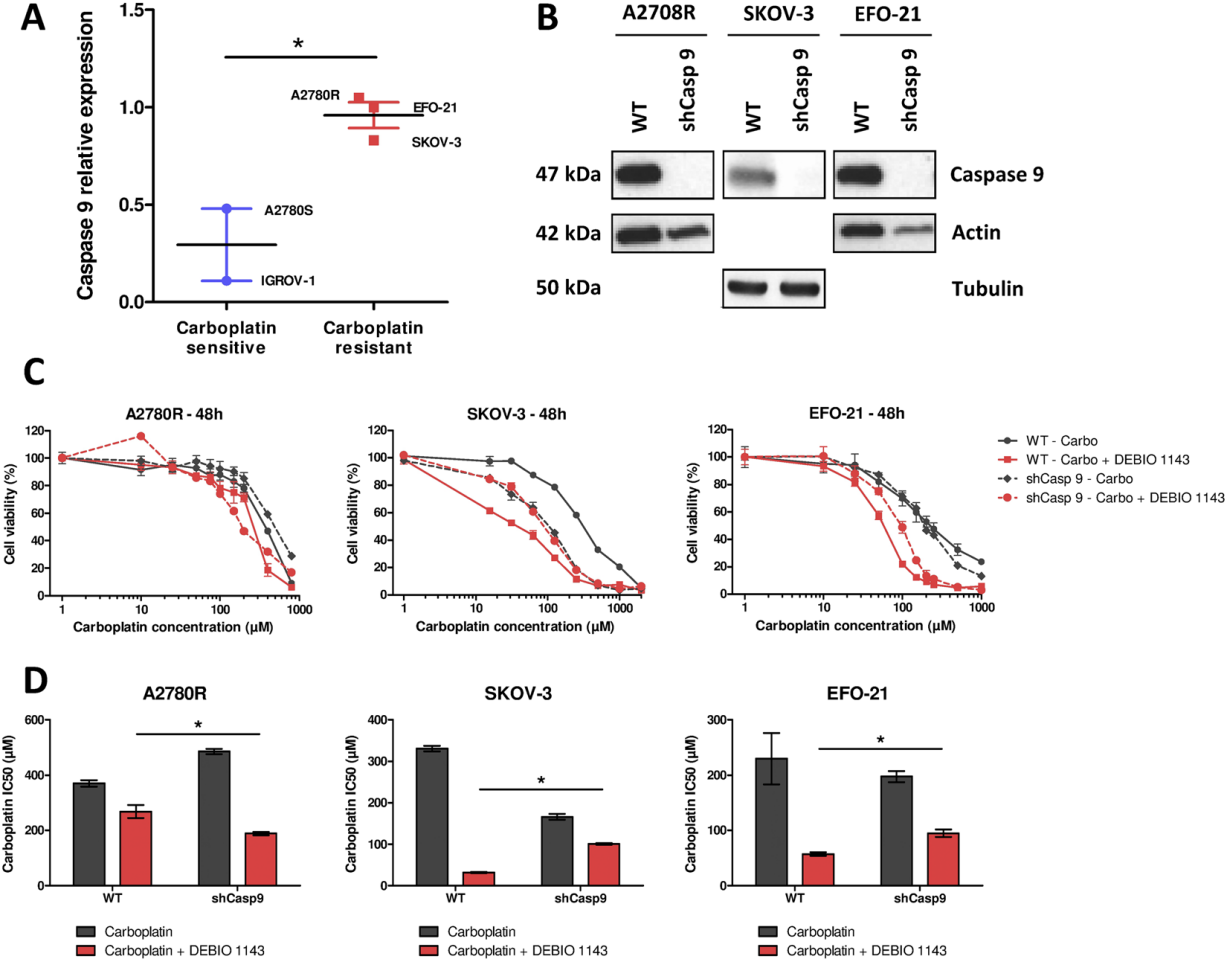
Role of caspase 9 expression in HOAC in response to DEBIO 1143. (**A**) Caspase 9 relative expression was determined in all cell lines by qPCR using GUSB, RPL19, TBP and TUBB as housekeeping genes (mean expression with EFO-21 caspase 9 expression as 1, *p < 0.05). (**B**) Protein extract from HOACs (A2780R, SKOV-3, and EFO-21 either control or following inhibition of caspase 9 expression) were analyzed by Western blots for the expression of caspase 9. The cells were treated with increasing concentrations of carboplatin alone (from 1000 µM to 15.625 µM by 2-fold dilution steps) or in combination with DEBIO 1143 (10 µM) 24 h after seeding. WT stands for wild-type. (**C**) 48 h after treatment, cell viability was determined by a colorimetric assay using WST-1. The negative control (non-treated) of each condition corresponds to the 100% cell viability. (**D**) The IC50 of carboplatin after 48 h of treatment was determined for each cell line (Mean +/− SEM, *p < 0.05, n = 4).

Depending on the cell lines, DEBIO 1143 had little to no effect on XIAP levels. As XIAP is able to interact with caspase 9, we used qPCR to assess the expression level of the latter in HOAC. Caspase 9 expression was approximatively three times higher in carboplatin-resistant HOAC compared to carboplatin-sensitive cells (Fig. 5A). We next wondered whether caspase 9 could be involved in DEBIO 1143 sensitivity. For this purpose we inhibited its expression in the three carboplatin-resistant cell lines (A2780R, EFO-21, and SKOV-3) using a LV encoding a caspase 9 shRNA. Marked inhibition of caspase 9 expression was achieved in all cell lines (Fig. 5B). Both the parent and genetically modified cell lines were then treated with carboplatin alone or in combination with DEBIO 1143 to determine the impact of caspase 9 on cell viability (Fig. 5C,D). When caspase 9 expression was down-regulated in A2780R cells, DEBIO 1143 induced an increased in carboplatin sensitivity (IC50 decreased by 37%). In contrast, the same caspase 9 down-regulation had the opposite effect when SKOV-3 and EFO-21 cells were treated with the carboplatin/DEBIO 1143 combination (IC50 increased by 320% and 167%, respectively) indicating that the expression of caspase 9 does not constitute a robust marker of DEBIO 1143 sensitivity.

### DEBIO 1143 alone, or in combination with carboplatin, strongly inhibits tumor growth in *in vivo* SKOV-3 s.c. and i.p. tumors models

**Figure 6:**
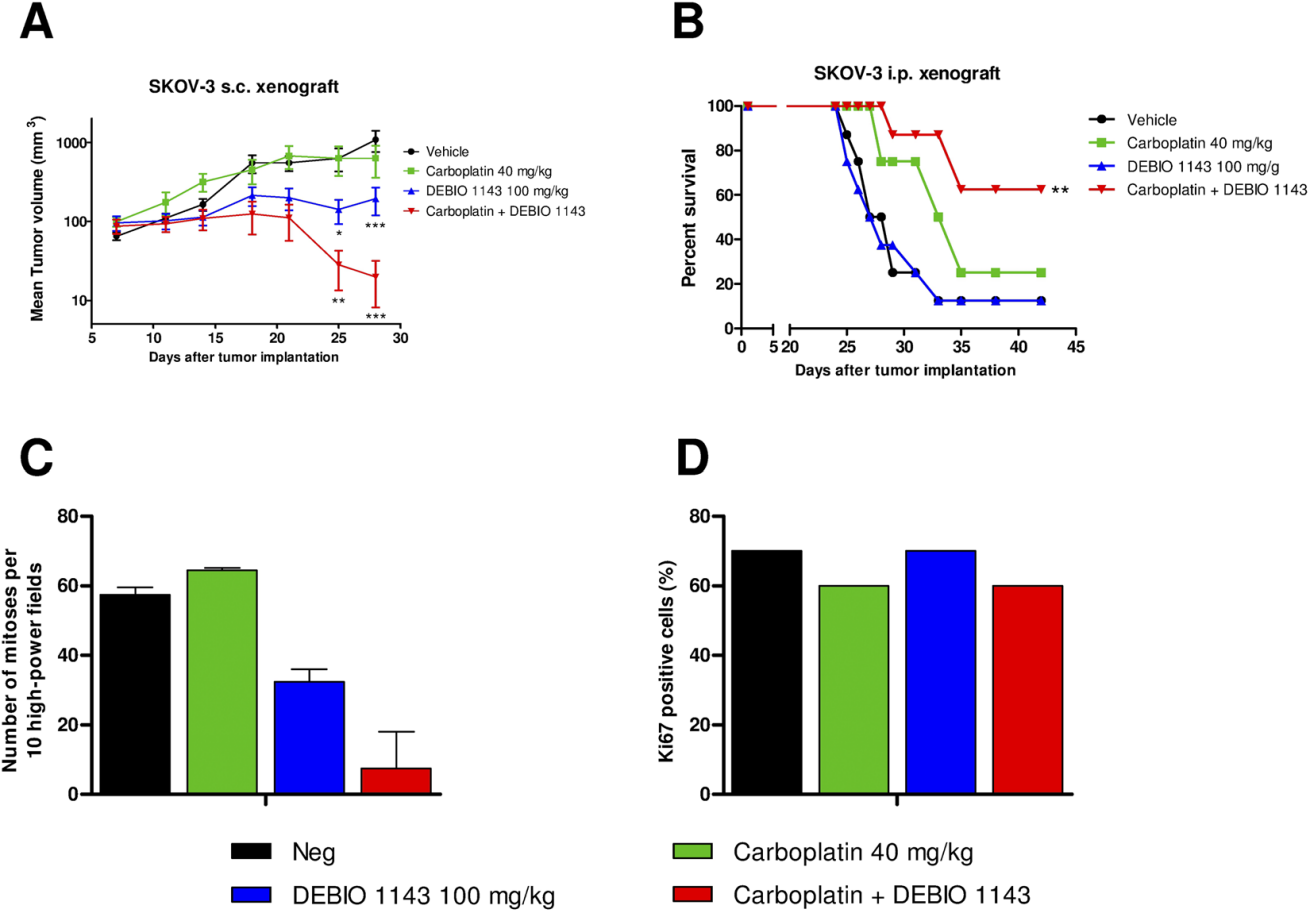
*In vivo* anti-tumor activity of DEBIO 1143, alone or in combination with carboplatin, in SKOV-3 tumor xenografts. Nude mice were engrafted (**A**) subcutaneously with 10^7^ SKOV-3 cells or (**B**) intraperitoneally with 25.10^7^ SKOV-3 cells. One week after the injection, they were treated intraperitoneally, once a week for three weeks with 40 mg/kg of carboplatin or PBS and treated by oral gavage, five days per week for three weeks with 100 mg/kg of DEBIO 1143 or vehicle. (**A**) Tumor volume was extrapolated from the measurement of tumor length and width twice a week. (Mean tumor volume +/− SEM, two-way ANOVA group comparison with the vehicle group, *p < 0.05, **p < 0.01, ***p < 0.001, n = 7 per group). (**B**) Survival curves of treated mice (Mann-Withney analysis in comparison with the vehicle group, **p < 0.01, n = 8 per group). (**C**) Number of mitoses per 10 high-power fields and (**D**) the percentage of Ki67 positive cells were quantified histologically on tumors three days after the last treatment. (Mean +/− SEM, n = 2 per group).

The carboplatin/DEBIO 1143 combination was assessed *in vivo* in s.c. SKOV-3 tumor xenografts in nude mice. Seven days after tumors were established, mice were treated intraperitoneally with 40 mg/kg of carboplatin once a week for three weeks and by oral gavage with 100 mg/kg DEBIO 1143 five days per week for three weeks (Fig. 6A). Two tumors per group were analyzed by histology three days after the last treatment.

Carboplatin treatment did not affect SKOV-3 tumor growth, confirming the carboplatin-resistant status of these tumors. In contrast to the *in vitro* results, DEBIO 1143 alone was able to inhibit tumor growth, with a slowdown in tumor growth observed in three mice and a complete regression in one mouse (out of seven). The combination of carboplatin and DEBIO 1143 triggered a strong tumor growth inhibition with slowdown of tumor growth in two mice, a complete regression in five mice (out of seven) and an apparent decreased number of mitoses per cell (Fig. 6C) despite no apparent change in the percentage of Ki67-positive cells (Fig. 6D).

As ovarian cancer routinely metastasizes, we assessed the *in vivo* efficacy of the same treatment regimen as used in the s.c. model in a mouse model in which SKOV-3 cells are injected intraperitoneally. This induces a very rapid dissemination of tumor cells and a carcinomatosis associated with ascites production within for weeks. Treatment started seven days after tumor cell injection and mice were sacrificed when moribund. Data were assessed using a time-to-endpoint analysis and showed that DEBIO 1143 alone did not affect SKOV-3 tumor growth. As expected, carboplatin treatment has little effect on tumor growth and mice presented with ascitic fluid. The combination of carboplatin and DEBIO 1143 triggered a strong inhibition of tumor growth, with any signs of tumor progression absent in more than 60% of mice and a significantly increased survival (p < 0.01, Mann-Whitney test, Fig. 6B).

## Discussion

Dysregulation of apoptotic pathways is a major contributor to cancer development and progression and plays a key role in cancer resistance to chemotherapy, targeted therapies and radiation. Even if tumors initially respond to these treatments, they often acquire resistance during the course of treatment. A recent study performed by Tamm *et al*.^35^ examined the expression of several members of the IAP family in the well-characterized NCI panel of 60 human tumor cell lines, correlating their expression at either the mRNA or protein levels with other tumor-related genes and with *in vitro* chemosensitivity data for 30,000 compounds. They concluded that analysis of IAP-family proteins may provide predictive information about responses to chemotherapy and survival for at least some subgroups of patients with acute myeloid leukemia (AML). Concerning ovarian cancer, Kleinberg *et al*. reported that XIAP and survivin are up-regulated in, respectively, effusions and metastasis of ovarian cancers, and could be considered to be targets and predictors of the patient outcomes^36^. Moreover, IAPs are frequently dysregulated in ovarian cancer and have been described as major regulators of the major regulators of TNF-α expression (TNF-α being a highly elevated cytokine in this cancer). In the presence of cIAPs, TNF-α activates prosurvival NF-κB signaling upon binding to its receptor. In the absence of cIAPs, however, TNF-α activates caspase 8 and extrinsic apoptosis. Thus, exploiting IAP-caspase interactions may be a logical strategy for anticancer drug development, especially in ovarian cancer^12,29,37^. SMAC mimetics could be used to increase the efficacy of standard therapies to exploit synergistic lethality. Reviews of the literature by Fulda^38^ or Philchenkov^39^ present the various preclinical studies detailing synergistic drug interactions of SMAC mimetics with a variety of cytotoxic therapies, such as chemotherapeutic drugs in solid tumors (paclitaxel, carboplatin, cisplatin, daunorubicin, etc) or in hematologic malignancies^40,41^, or targeted therapies such as TRAIL receptor agonists^42^, epigenetic drugs, or immunotherapies^43,44^. Several SMAC mimetics have been evaluated in early clinical trials as monotherapy for the treatment of solid tumors and hematologic cancers, and multiple clinical trials are currently ongoing, mainly in the combination setting^38,45,46^.

Notably, DEBIO 1143 has been tested as monotherapy for the treatment of solid tumors and lymphoma in a phase 1 clinical trial (NCT01078649), as well as in patients with high-risk AML combined with daurorubicin and cytarabine (NCT01265199^31^). Currently, DEBIO 1143 is being evaluated in a multicenter, double-blind, randomized, placebo-controlled phase II study aiming to assess the antitumour activity of combined paclitaxel and carboplatin with and without DEBIO 1143 at the end of neoadjuvant treatment (prior to interval debulking surgery) in patients with newly diagnosed EOC (EudraCT Identifier 2015-005137-42). This trial was triggered by a positive efficacy signal in patients with advanced EOC in a phase I trial (NCT01930292^32^). Furthermore, DEBIO 1143 is also under assessment in a multi-center phase I/II trial in combination with concurrent chemoradiotherapy in patients with previously untreated locally advanced squamous cell carcinoma of the head and neck (NCT02022098^47^). DEBIO 1143 is also being assessed in a multi-center, open-label phase-Ib study evaluating the combination with the anti-PD-L1 antibody avelumab in patients with advanced solid malignancies and, in an expansion cohort, in patients with locally advanced or metastatic non-small cell lung cancer (NCT03270176).

Apart from DEBIO 1143, several other SMAC mimetic compounds are currently being investigated in active clinical trials (www.clinicaltrials.gov46). The bivalent SMAC mimetic birinapant is currently being tested in combination with the anti-PD1 immune checkpoint inhibitor pembrolizumab (NCT02587962). In addition, notably, a study in combination with carboplatin in recurrent high grade ovarian, fallopian tube, or primary peritoneal cancer is awaiting recruitment start (NCT02756130). LCL161 monotherapy is under assessment in a phase II trial in patients with different forms of myelofibrosis, as well as in various oncology indications in combination with other drugs such as topotecan, cyclophosphamide, the anti-PD-1 antibody PDR001, or the anti-IL17 antibody CJM112 (NCT02649673, NCT01955434, NCT03111992, NCT02098161, NCT02890069). Of note, the topotecan study includes patients with select gynecologic malignancies including ovarian cancer. More recently, a series of compounds has also entered clinical testing. These include the compound ASTX660, which is being tested as monotherapy in a phase I/II trial in patients with solid tumors and lymphomas (NCT02503423). Furthermore, a trial is underway testing BI 891065 alone and in combination with the anti-PD-1 antibody BI 754091 in patients with incurable or metastatic tumors (NCT03166631). Finally, another bivalent compound, APG-1387, is undergoing assessment in a trial in advanced solid tumors and lymphomas.

For the clinical development of all SMAC mimetics it would be valuable to have biomarkers able to predict patient sensitivity. Clinical trials have evaluated several markers of target engagement in surrogate tissue such as peripheral blood mononuclear cells (PBMC) or tumor tissue (e.g., degradation of cIAP1 levels due to SMAC mimetics), markers of apoptosis in blood or tumor tissue (e.g., cleavage of caspases or caspase substrates such as PARP or cytokeratin-18), and blood markers of inflammation related to SMAC mimetic-mediated activation of signaling pathways (e.g., increases in inflammatory cytokines such as TNF-α, MCP-1, IL-6, and IL-8)^38^. Focusing on clinical trials involving DEBIO 1143, Dipersio *et al*. revealed that responders more frequently showed plasma increases of TNF-α and IL-8 post-first dose of DEBIO 1143 during AML treatment (when patients were treated with a combination of DEBIO 1143, daunorubicin, and cytarabine)^48^. There was no obvious correlation between changes in cIAP1 and types of AML or treatment success. By contrast, neither an increase in plasma IL-8 nor any correlation with DEBIO 1143 exposure was observed by Hurwitz *et al*. in the clinical trial that involved patients with advanced cancers^31^. To date, no clear biomarkers of the sensitivity to DEBIO 1143 have been identified.

Here, we showed that DEBIO 1143 was able to sensitize carboplatin-resistant cell lines to carboplatin and to restore A2780R to the carboplatin-sensitivity of its parental cell line, A2780S. We wanted to determine which cell death pathway was activated by the combination of DEBIO 1143 with carboplatin. Overall, we observed no activation of apoptosis in so-called DEBIO 1143-resistant strains. However, the carboplatin/DEBIO 1143 combination seemed to increase, although not significantly, early apoptosis in A2780S cells, which may precede a longer-term effect on the lineage we would not have observed during 72-hour treatments. In the platinum-resistant cell lines A2780R and SKOV-3, the carboplatin/DEBIO 1143 combination resulted in an increase in the number of early apoptotic cells compared to monotherapy treatment. On the other hand, EFO-21 cells did not exhibit an induction of early apoptosis; these cells were, however, extremely sensitive to the combination of the two compounds from a cell viability point of view. This observation, corroborated by the fact that late apoptosis and necrosis was increased in this cell line when treated with the carboplatin/DEBIO 1143 combination, indicates that this treatment induces an apoptosis-independent cell death pathway in EFO-21 cells.

Necroptosis, a caspase-independent cell death pathway, has been reported to be potentially induced by SMAC mimetics in several cancers, including ovarian cancer^49–51^. Lecis and McCabe recently described in their respective works that SMAC mimetics can trigger necroptosis in a TNF-α dependent manner. This inflammatory process is mainly dependent on macrophage activity, potentially explaining why SMAC mimetics, in our case DEBIO 1143, are inactive as monotherapies *in vitro* but show antitumor activity alone *in vivo* in s.c. mouse models. To test the potential activation of necroptosis in EFO-21 cells, we pre-treated them with Z-Vad, an apoptosis inhibitor, or Necrostatin-1, a necroptosis inhibitor. Only Necrostatin-1 prevented the cell viability inhibition induced by DEBIO 1143 combined or not with carboplatin in EFO-21 cells, indicating that our SMAC mimetic activates necroptosis in this cell line. Such a sensitizing effect of a SMAC mimetic-containing combination treatment confirms findings from Bhatti *et al*., who showed that SMAC mimetics/Bortezomib co-treatment was synergistic and triggered cell death *via* necroptosis when apoptosis was blocked, or alternatively via both apoptosis and necroptosis^49^.

Here, we report that induction of necroptosis is an alternative strategy to trigger programmed cell death in apoptosis resistant ovarian cells. We demonstrate that the SMAC mimetic DEBIO 1143 induces necroptosis in ovarian cells in which apoptosis is blocked by the caspase inhibitor Z-Vad. This could be due to the production of TNF-α by ovarian cells, as Hannes *et al*. suggested in the case of pancreatic carcinoma cells^52^.

We demonstrated that caspase 9 was highly expressed in carboplatin-resistant ovarian cancer cell lines, but that this was independent of sensitivity to DEBIO 1143. This result, however, does not alter the crucial need to find markers of DEBIO 1143 sensitivity that will allow identification of the patients who could benefit from a carboplatin/DEBIO 1143 combination.

We showed that DEBIO 1143, a SMAC mimetic currently in clinical trials, is able to restore carboplatin sensitivity in ovarian cancer cells. The capacity to directly trigger apoptosis by inhibiting IAPs, or to induce necroptosis by an indirect action on the stroma, constitutes a new perspective to specifically treat patients with chemoresistant ovarian cancer. The discovery of this synergistic combination, that is effective even when apoptosis is blocked, has important implications for the development of new treatment strategies.

## Materials and Methods

### Drugs

Carboplatin was provided by the Claudius Regaud Institute pharmacy at a concentration of 26.9 mM. DEBIO 1143 was provided by Debiopharm International SA (Lausanne, Switzerland) and was diluted in dimethylsulfoxide to obtain a 100 mM stock solution.

### Cell culture

The human ovarian adenocarcinoma cell (HOAC) line SKOV-3 (ATCC® numbers HTB-77) was obtained from the American Type Culture Collection (Manassas, VA). The HOAC line IGROV-1 was a gift from the Institut Gustave Roussy, Villejuif. A2780S and platinum-resistant A2780R cells were obtained from Sigma Aldrich (France). The HOAC line EFO-21 was obtained from the Deutsche Sammlung von Mikroorganismen und Zellkulturen GmbH (DSMZ, Germany). HOAC were cultured in RPMI 1640 medium containing 10% fetal calf serum, supplemented with 1% L-Glutamine and 1% penicillin-streptomycin. Cell lines were maintained as monolayers at 37 °C in an humidified 5% CO2 atmosphere.

### shRNA-transduced cell line generation

pLKO-1® is a lentiviral vector (LV) encoding the shRNA (small hairpin RNA) under the control of the U6 promoter as well as the puromycin resistance gene under the control of the hPGK promoter (Sigma Aldrich, France). We constructed two different LVs corresponding to various targeted regions of caspase 9:

1: 5′CCGGCTTTGTGTCCTACTCTACTTTCTCGAGAAAGTAGAGTAGGACACAAAGTTTTT

2: CCGGCAGCTTCCAGATTGACGACAACTCGAGTTGTCGTCAATCTGGAAGCTGTTTTT

The same LV encoding a scramble shRNA (5′ TTCTAGAGATAGTCTGTACGTTTCAAGAGAACGTACAGACTATCTCTAGAAG) was used as negative control. 293 T cells were kindly provided by Genethon (France). Generation of 293T- pLKO-1 casp9 1, casp9 2, or control and preparation of high titer LV pseudotyped with VSV-G protein occurred as previously described. 50 × 10^3^ cells were plated on 35 mm dishes 24 h prior to transduction with viral vectors at a Multiplicity of infection (MOI) of 10:1. Genetically modified cells were selected through their resistance to puromycin at 1 µg/ml.

### Viability assay and IC50 determination

5 × 10^3^ HOAC (IGROV-1, A2780S, A2780R, SKOV-3 and EFO-21) were seeded in 96-well plates and treated with increasing concentrations of carboplatin (from 1000 µM to 15.625 µM by 2-fold dilution steps) or DEBIO 1143 (from 1 mM to 1 nM by log dilutions) or a combination of carboplatin (from 1000 µM to 15.625 µM by 2-fold dilution steps) and DEBIO 1143 (10 µM) 24 h after seeding. At 48 h or 72 h after treatment, cell viability was determined by a colorimetric assay using a tetrazolium salt, WST-1 (Roche). A non-linear regression was performed and the relative IC50 values were determined with GraphPad Prism.

### Annexin-V-FITC/Propidium Iodide (PI) assay

7.5 × 10^4^ HOAC (IGROV-1, A2780S, A2780R, SKOV-3 and EFO-21) were seeded in 12-well plates and treated with carboplatin (IC50 of each cell line after 48 h of treatment) or DEBIO 1143 (10 µM) or a combination of carboplatin and DEBIO 1143 24 h after seeding. One or two days later, cells were washed twice with fresh phosphate-buffered saline (PBS), trypsinized (supernatants are kept) and stained with a FITC-Annexin-V/PI apoptosis detection kit (BD Pharmigen) according to the manufacturer’s protocol. FITC-Annexin-V staining and PI incorporation were measured in cells with a FACS Calibur flow cytometer and analyzed with Cell Quest software.

### Western blot (PARP, cIAP1, XIAP, caspase 9)

25 µg of proteins were separated by SDS-PAGE on a 10% polyacrylamide gel. Proteins were transferred to a polyvinylidene difluoride membrane. Membranes were saturated for 1 h in tris-buffered saline (TBS) (50 mM Tris, 150 mM NaCl)/0.1% Tween-20/5% milk and incubated overnight at 4 °C with a rabbit primary antibody directed against: PARP (polyclonal, 1:1000, Cell Signaling), XIAP (monoclonal, 1:1000, Cell Signaling), cIAP1 (1:1000, Abcam) or caspase 9 (1:1000, Abcam). Membranes were washed three times with TBS/0.1% Tween 20 (TT) then incubated for 1 h 30 with a secondary antibody anti-rabbit (1:2000, Cell Signaling) coupled with HRP (horse raddish peroxydase). Membranes were washed three times with TT. Immunocomplexes were revealed by enhanced chemiluminescence (GE Healthcare, Amersham) and visualized with a photon camera (ChemiDoc, BioRad). Picture analyses were realized with Image Lab.

### Apoptosis and necroptosis inhibition

7.5 × 10^4^ HOAC (SKOV-3 and EFO-21) were seeded in 12-well plates. At 48 h after seeding, cells were pre-treated 2 h or not with Z-Vad 20 µM (pan-caspase inhibitor, Switzerland) or necrostatin-1 50 µM (inhibitor of RIPK1 and 3, R&D systems, France) then treated with DEBIO 1143 (10 µM) or a combination of carboplatin (IC50 of each cell line after 48 h of treatment) and DEBIO 1143 (10 µM). At 24 h after treatment, cell viability was determined by a colorimetric assay using a tetrazolium salt, WST-1 (Roche).

### RNA extraction and quantitative PCR

Total RNA was isolated using the miRNeasy Mini Kit (Qiagen) and 600 ng RNA wasreverse transcribed using Transcriptor Universal cDNA Master (Roche Diagnostics GmbH, Germany) according to the manufacturer’s instructions. Quantitative PCR was carried out using LightCycler® DNA Probes Master including Taqman probes (Roche Diagnostics GmbH, Germany) to amplify human caspase 9. RPL19, TUBB, GUSB, and TBP were included as housekeeping genes. Expression levels of caspase 9 were reported as the mean ΔCT of caspase 9 to each housekeeping gene. The comparison of caspase 9 expression among all cell lines was realized using ΔΔCT calculated relative to EFO-21 cells. Relative expression was reported considering the EFO-21 caspase 9 expression to be 1.

### Animals

Four- to five-week-old female Swiss Nude athymic mice (Charles River laboratories, France) were included after approval from the Claudius Regaud Institute animal ethics committee (ICR-2014-002). They were housed according to European Laboratory Animal Science Association standards. All experiments were performed and approved in accordance with European guidelines and regulation. They have been approved by the French ethic committee (national agreement) and by a local committee (protocol number ICR-2014-002). Mice experimentations began after one week of quarantine.

### *In vivo* SKOV-3 subcutaneous (s.c.) xenografts

10^7^ SKOV-3 cells were implanted subcutaneously into one flank of seven mice per subgroup. After seven days, mice were treated intraperitoneally, once a week for three weeks, with 40 mg/kg of carboplatin or PBS, and treated by oral gavage, five days per week for three weeks, with 100 mg/kg of DEBIO 1143 or vehicle (malic acid/sodium acetate buffer). The well-being and body weight of mice were checked every two to three days. Tumor lengths (L) and widths (w) were measured twice a week and the tumor volume extrapolated using the formula: (L × w²)/2. Mice were sacrificed *via* cervical dislocation 28 days after the injection of tumor cells. Tumors were sampled then conditioned for histological analysis (formol fixation).

### *In vivo* SKOV-3 intraperitoneal (i.p.) xenografts

2.5 × 10^7^ SKOV-3 cells were intraperitoneally implanted into eight mice per subgroup. After seven days, mice were treated intraperitoneally, once a week for three weeks, with 40 mg/kg of carboplatin or PBS and treated by oral gavage, five days per week for three weeks with 100 mg/kg of DEBIO 1143 or vehicle (malic acid/sodium acetate buffer). The well-being and body weight of mice were checked every two to three days. Mice were sacrificed *via* cervical dislocation 21 days after the injection of tumor cells.

### Histological analysis

Proliferative indices of tumor from s.c. injections were assessed by immunohistochemical staining of paraffin-embedded tumor sections for the proliferation marker Ki67 using mouse monoclonal MIB1 antibody. Ki67-positive nuclei were counted in random fields. The mitotic index was assessed by evaluating the number of cells in mitosis per high-power field (10 high-power fields per tumor).

### Statistical analysis

For this entire study, statistical significance was set at p < 0.05. Group comparisons were made using the Student’s t-test and Mann-Whitney test (parametric data). Multiple group comparisons were made using two-way ANOVA. Each *in vitro* experiment was performed at least three times.

## Electronic supplementary material

Supplementary Dataset 1
