# Supplementary material for: DEBIO 1143, an IAP inhibitor, reverses carboplatin resistance in ovarian cancer cells and triggers apoptotic or necroptotic cell death
Source: Sci Rep. 2018 Dec 14;8:17862. doi: 10.1038/s41598-018-35860-z (PMC6294826; doi:10.1038/s41598-018-35860-z)
Supplement: Supplementary file 1 — Supplementary Dataset 1 [file 41598_2018_35860_MOESM1_ESM.pdf]

DEBIO 1143, an IAP inhibitor, reverses carboplatin resistance in ovarian cancer cells and triggers apoptotic or necroptotic cell death

Benoît Thibault, Ludivine Genre, Augustin Le Naour, Clothilde Broca, Eliane Mery Grégoire Vuagniaux, Jean Pierre Delord, Norbert Wiedemann, Bettina Couderc

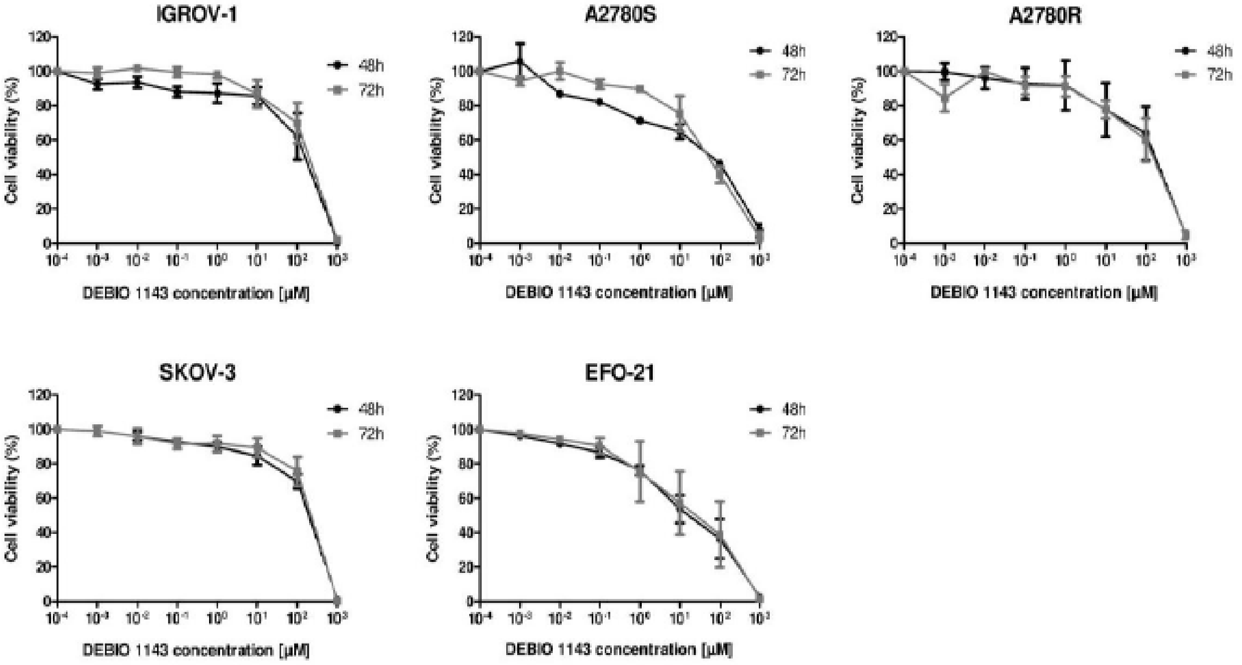

Supplementary Figure 1. *In vitro* cytotoxic effects of DEBIO 1143 as monotherapy. HOAC (OVCAR-3, IGROV-1, A2780S, A2780R, SKOV-3, and EFO-21) were treated with increasing concentrations of DEBIO 1143 alone (from 1000 µM to 1 nM by 10-fold dilution steps) 24 h after seeding. 48 h or 72 h after treatment, cell viability was determined by a colorimetric assay using WST-1. The negative control (non-treated) of each condition corresponds to the 100% cell viability. (Mean +/- SEM, n=3).

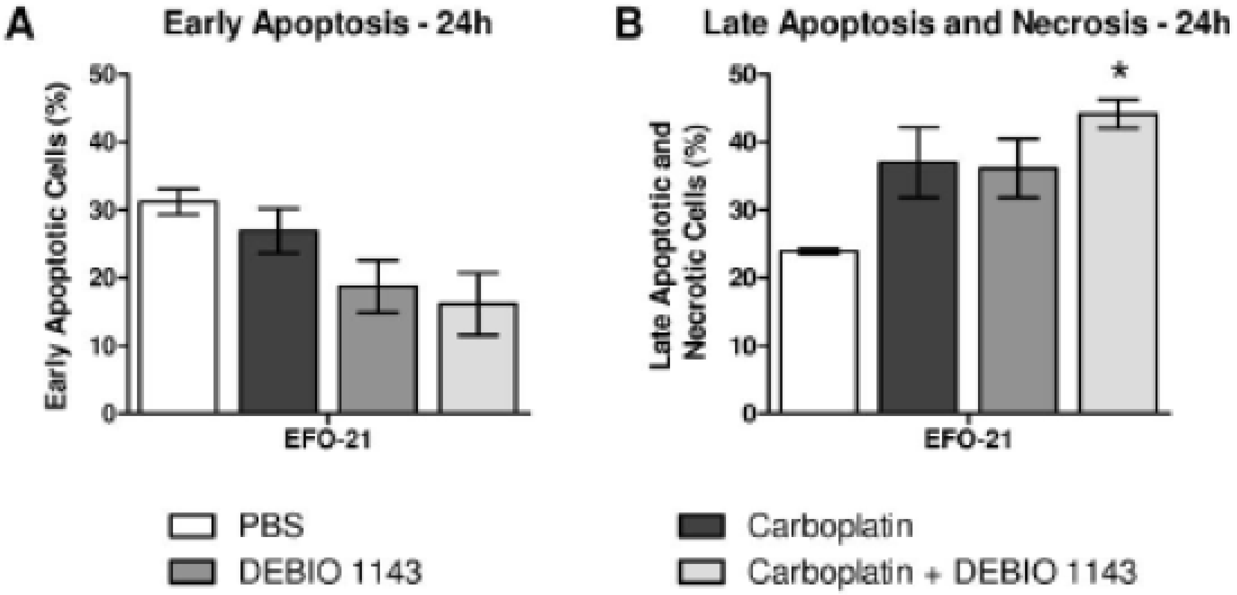

Supplementary Figure 2. *In vitro* induction of apoptosis in EFO-21 cells by DEBIO 1143 alone or in combination with carboplatin. EFO-21 cells were treated with carboplatin (IC50 after 48 h of treatment), DEBIO 1143 (10 µM) or a combination of both treatments. The negative control corresponds to non-treated cells. (A) and (B) 24 h after treatment, cells were stained with a FITC-Annexin V/PI apoptosis detection kit. FITC-Annexin staining and PI incorporation were measured in cells with a FACS Calibur flow cytometer and analyzed with Cell Quest software. (A) Early apoptotic cells correspond to the Annexin V positive and PI negative population. (Mean +/- SEM, * = $p<0.05$, $n=3$). (B) Late apoptotic and necrosis cells correspond to the Annexin V positive and PI positive population. (Mean +/- SEM, * = $p<0.05$, $n=3$).
